# Nonlinear Refraction and Absorption in Polymers Used for Femtosecond Direct Laser Writing

**DOI:** 10.1021/acsomega.4c09152

**Published:** 2024-12-24

**Authors:** Renan Cunha, João V. P. Valverde, Leonardo De Boni, Lino Misoguti, Cleber Renato Mendonça

**Affiliations:** Instituto de Física de São Carlos, Universidade de São Paulo, São Carlos, SP 13560-970, Brasil

## Abstract

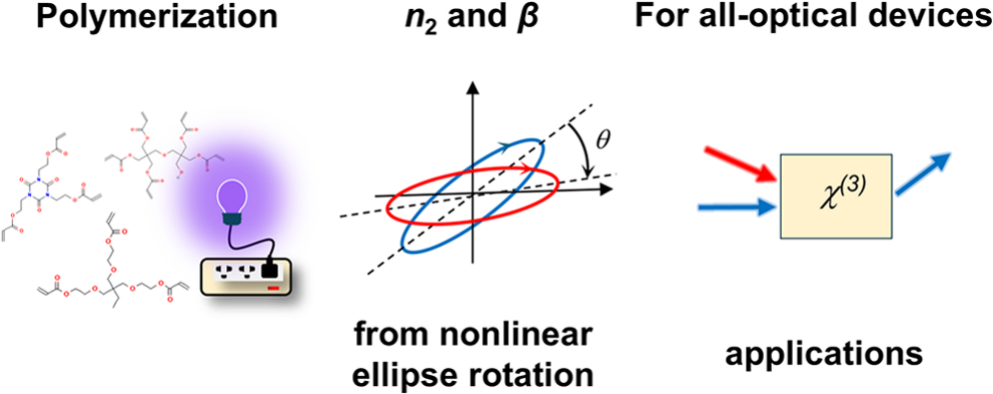

Direct laser writing (DLW) has been recognized as a unique technique for three-dimensional (3D) prototyping with resolution beyond the diffraction limit. One trend in DLW technologies is the use of polymers, given their favorable mechanical properties and optical quality, rendering them promising for the next generation of nonlinear photonic devices. However, absorptive properties that facilitate DLW processes may also hinder the performance of polymers as all-optical devices. Furthermore, the increasing use of ultrashort pulse lasers makes refractive index dispersion a factor of fundamental importance in studying candidate materials for broadband applications. Here, we study the suitability of resins commonly used in DLW for all-optical broadband applications. We provide linear and nonlinear refractive and absorptive spectra of acrylate-based resins with different compositions, and IP-S and IP-Dip from Nanoscribe. We found nonlinear refractive indices four times higher than reported values for fused silica, one of the most widely used photonic materials, and we evaluate the potential of these materials using group velocity dispersion and figures of merits as comparative metrics.

## 1 Introduction

The rapid evolution of photonics has borne witness to remarkable advancements, driving applications from life sciences to next-generation devices.^1−4^ One notable technique embraced by the photonics community is direct laser writing (DLW).^5−7^ As a tool for fabricating structures at the microscopic scale, this technique has facilitated innovations in numerous fields, from quantum science to artificial intelligence.^8−13^ A prolific trend that has garnered attention is the utilization of organic resins as printing material in DLW by multiphoton polymerization.^7,14−21^ The molecular attributes of organic compounds allow for the fine-tuning of mechanical and nonlinear optical properties, thus enabling a plethora of novel applications. These include scaffolds for cell culture, biomedical implants, soft robotics, microlens, paper-based devices for flexible electronics, microresonators for nonlinear photonic devices, to name a few.^22−29^

Most of the previous examples employ ultrashort pulse lasers to achieve the desired printing resolution of photonic devices. In some cases, these lasers are also required for the operation of devices, such as in all-optical switches and modulators. As the spectrally broad pulses of such lasers travel through the medium, they become highly susceptible to dispersion. Moreover, the usual high intensities render the propagation of ultrashort pulses prone to nonlinear refractive dispersion.^30^ These high intensities in the medium produce propagation self-effects, causing the refractive index experienced by the incident beam to have an intensity-dependent component.^30^ While it is desirable to find materials with high nonlinearities for photonic devices, these effects may constrain the versatility of organic materials when their optical Kerr response is strongly dependent on the excitation wavelength. Also, organic materials commonly used for three-dimensional (3D) prototyping photonic devices are designed to facilitate DLW processes, which rely heavily on multiphoton absorption processes, including two-photon absorption. Losses associated with two-photon absorption can become a significant limitation due to the intensity-dependent absorption. Therefore, selecting materials for broadband photonic applications requiring significant third-order effects and well-behaved dispersion can only be accomplished through rigorous characterization of the dispersion curves for the linear, *n*_0_, and nonlinear, *n*_2_, refractive indices, as well as linear and nonlinear absorption.

There is a broad range of approaches for extracting *n*_2_ of materials, with the standard *Z*-scan technique being one of the most popular.^31^ The ease of implementation and precision are two main reasons for its popularity. However, the technique carries certain drawbacks, such as its strong dependence on the incident beam profile, sensitivity to sample inhomogeneities, and difficulty in measuring local nonlinear refraction. Some of these drawbacks are associated with the dependence of *n*_2_ on the magnitude of the transmittance variation. Recently, our group reported the determination of *n*_2_ through accurate measurements of the nonlinear ellipse rotation (NER) effect.^32^ The NER technique implements a transmittance-based approach, keeping the same advantages of the *Z*-scan. At the same time, the extraction of *n*_2_ from NER is based on phase measurements rather than magnitude.

Here, we employed NER to evaluate the suitability of polymers commonly used in DLW for all-optical broadband applications based on a comparison of their *n*_0_ and *n*_2_ spectra against their linear and nonlinear absorption. We studied acrylate-based samples with different resin compositions and IP-S and IP-Dip (Nanoscribe). The *n*_2_ values thus obtained are about four times higher than reported values for fused silica, one of the most widely used photonic materials. The polymers also show well-behaved refractive and absorptive spectra, highlighting the promising potential of organic materials in third-order effect-based technologies. Finally, we use the group velocity dispersion and figures of merit as comparative metrics for all the samples.

## 2 Experimental Section

### 2.1 Sample Preparation

We investigated eight samples that can be broadly divided into two groups: (I) six acrylate-based samples that received the addition of a photoinitiator in their composition and (II) two samples whose compositions are already commercially available with photoinitiators. For group I, we labeled the six samples according to the proportion of monomers used in their composition. We prepared combinations of the monomer tris(2-hydroxyethyl)isocyanurate triacrylate (Sartomer 368, SR 368) with monomers etoxilated(6)trimethylolpropane triacrylate (Sartomer 499, SR 499) or dipentaerythritol pentaacrylate (Sartomer 399, SR 399), so that the compositions had decreasing amounts of the SR 368 monomer as shown in Table 1.

**Table 1:** Composition of Acrylate Samples

sample label	SR 368 (wt %)	SR 499 (wt %)	SR 399 (wt %)
SR1	100	0	0
SR2	90.04	9.95	0
SR3	69.97	30.03	0
SR4	50	50	0
SR5	10.26	0	89.74
SR6	0	0	100

Each composition received the addition of 3 wt % (based on total mass) of the photoinitiator ethyl-2,4,6-trimethylbenzoyl phenylphosphinate (commercially known as Lucirin TPO-L) and was subjected to magnetic stirring at 40 °C until achieving homogenization. During a stirring process, we used a cover to avoid exposing the samples to ambient light. The resulting mixture was poured into a mold with a defined thickness and ultraviolet (UV) irradiation was then employed to cure samples completely, resulting in solid polymers with an average thickness of 1.8 mm. We used a lamp with UV emission between 300 and 450 nm and exposed the samples to an intensity of 4 mW/cm^2^ for 60 min. At the midpoint of the total UV light exposure time, each sample was flipped to ensure more homogeneous curing.

Group II comprises two samples: IP-S and IP-Dip (Nanoscribe, Inc.). Both received UV doses until the complete curing. We used the 325 nm line of a CW He–Cd laser as the UV light source, exposing the samples to an intensity of 9 mW/cm^2^ for 180 min. Here as well, each side of the sample was subjected to half of the total exposure time to ensure more homogeneous curing. This process produced solid samples of 1.8 mm for IP-S and 0.5 mm for IP-Dip.

### 2.2 Linear Optical Properties

We measured the linear optical properties of all the investigated materials using an Abbe refractometer (Pulfrich PR2, Carl Zeiss/Jena) with a GoF4 prism and 1-Bromonaphthalene, 97% (Aldrich Chemical Co., Inc.) as index matching fluid and a Shimadzu UV–vis 1800 spectrophotometer providing absorption spectra in the 190–1100 nm range.

### 2.3 Nonlinear Optical Properties

We used the NER method to determine the nonlinear refractive index of the samples. The NER effect is akin to the optical activity effect, where light-matter interactions cause the polarization plane of the incident light beam to rotate as the beam travels through the material. The nonlinear ellipse rotation is a Kerr-type effect in which an intense elliptically polarized beam has its major axis direction rotated by an angle θ related to the third-order susceptibility of the material,^33^ χ^(3)^. Since χ^(3)^ is related to *n*_2_ through *n*_2_ = 3*Re*{χ^(3)^}/4ε_0_*n*_0_*c*, where ε_0_ is the vacuum permittivity, *n*_0_ is the linear refractive index, and *c* is the speed of light, measuring θ allows the determination of *n*_2_. For isotropic media, only two independent components of χ^(3)^ are nonzero.^32,33^ In this case, the susceptibility tensor can be written as χ^(3)^*= A*/3 *+ B*/6, where *A* = χ_1122_ and *B* = χ_1221_. Measurements of the ellipse rotation allow access only to component *B*.

An accurate method for measuring the NER angle, also in thick samples, has recently been reported.^32,34^ In this method, a linear polarizer and a quarter-wave plate (λ/4) prepare the initial polarization state, while a rotating polarizer at the detection modulates the signal as a cosine squared waveform according to Malus’ law. The maximum (minimum) transmittance indicated by the modulated signal occurs when the fast axis of the rotating polarizer coincides with the major (minor) axis of the ellipse. Hence, a beam subjected to NER will display an identical cosine waveform to the beam where NER is absent, albeit with a phase shift. Since the NER effect is only non-negligible at the focus in thick samples, the phase shift of the waveform as the sample approaches the focus can be measured using a dual-phase lock-in amplifier (LIA). This configuration allows the sample (polymers) and the reference (fused silica) to be measured simultaneously under the same experimental condition.

For laser pulses with a temporal and spatial Gaussian profile, the time-averaged NER angle α as a function of position *z* in thick samples measured by the LIA is given by^35^
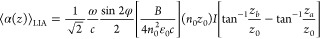
1in which ω is the excitation frequency, *c* is the speed of light, φ is the angle between the polarization plane of the incident beam and the quarter-wave plate fast axis, *n*_0_ is the linear refractive index, ε_0_ is the vacuum permittivity, *z*_0_ is the Rayleigh length, *I* is the laser irradiance, *z*_*b*_ = *z* + *L*/2*n*_0_ and *z*_*a*_ = *z* – *L*/2*n*_0_, where *L* is the sample thickness, and *B* = *8n*_2_*n*_0_^*2*^*ε*_0_*c*/3. Expression (1) demonstrates how acquiring the NER angle allows for the precise determination of *n*_2_.

**Figure 1:**
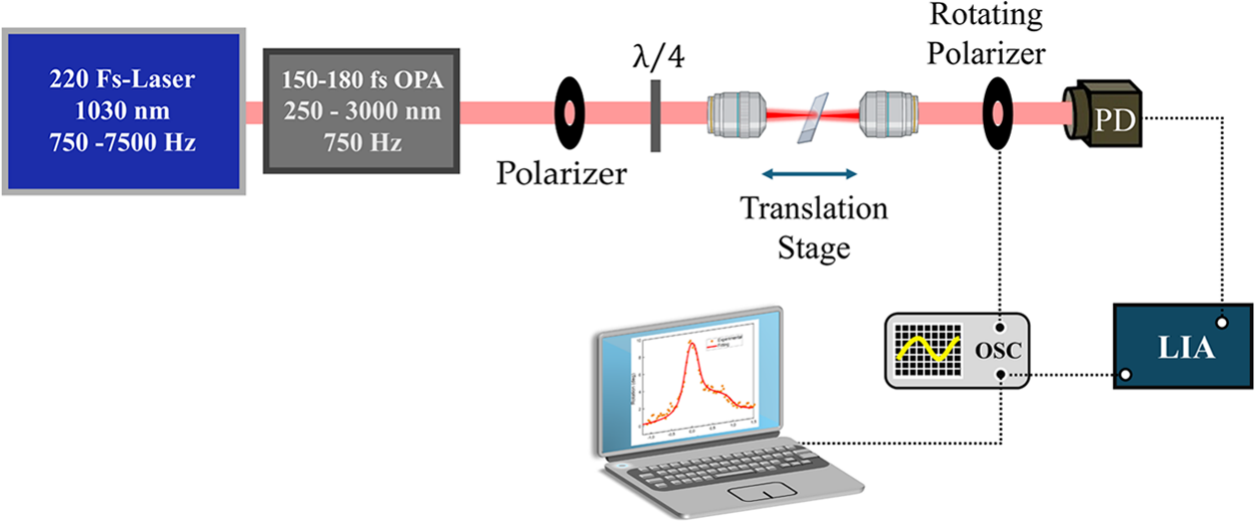
Experimental setup for accurate measurements of the NER angle.^36^ The Polarizer and the quarter-wave plate (λ/4) prepare elliptical polarization states. Two objective lenses focus and collimate the beam interacting with the sample moving in a Translation Stage. The Rotating Polarizer, the photodiode (PD), the dual-phase lock-in amplifier (LIA), and the oscilloscope (OSC) constitute the detection that produces the representative NER spectrum seen in the computer screen and in Figure 4.

The setup we used for determining the nonlinear dispersion using NER is illustrated in Figure 1. An amplified Yb:KGW laser (wavelength: 1030 nm, pulse duration: 220 fs–Pharos PH1, Light Conversion) pumps an optical parametric amplifier (Orpheus, Light Conversion), serving as a tunable excitation source in the 250–3000 nm range, with pulse duration of 150–180 fs. A linear polarizer and λ/4 plate prepare the incident polarization of the beam, which interacts with the sample through a 3× objective. The second objective collimates the beam and directs it toward a rotating polarizer and a photodiode connected to a dual-phase lock-in amplifier and an oscilloscope. The technique does not require any special sample preparation, and here we mounted each sample on a 1 mm-thick fused silica substrate, used as a reference. We attached both to a holder on the translation stage, allowing the signal from each sample and the silica to be obtained simultaneously, ensuring the same experimental conditions.

Since the technique works as an open aperture *Z*-scan, it allows for the simultaneous determination of nonlinear absorption. When a beam with sufficiently high intensity *I* interacts with a medium, it induces intensity-dependent absorption, α = α_0_ + β*I*. In this case, the absorption coefficient α has a linear component α_0_ corrected by a nonlinear component β. The contribution of the two-photon absorption coefficient β can be calculated through
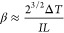
2where Δ*T* is the normalized transmittance variation and *L* is the sample thickness. For tightly focused excitation, *L* = *n*_0_*z*_0_ is the effective thickness of the sample.

## 3 Results

**Figure 2:**
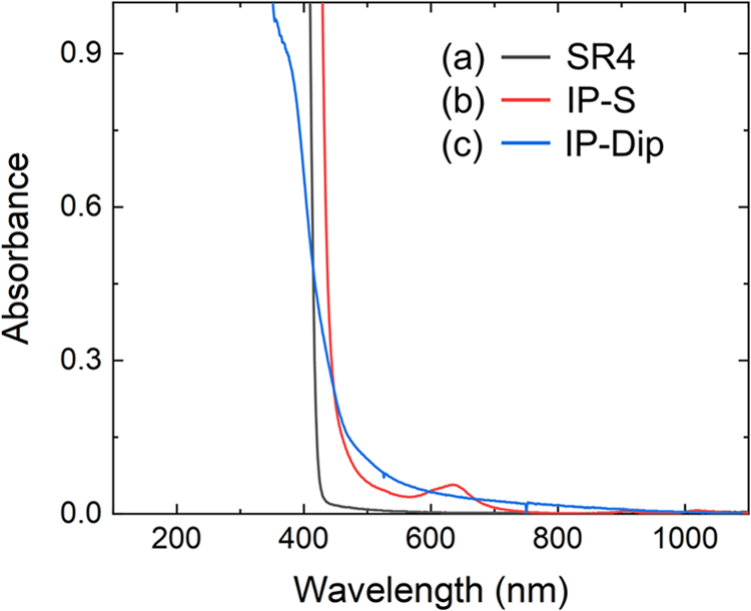
UV–vis absorption spectra of completely photopolymerized samples (a) SR4, (b) IP-S, and (c) IP-Dip.

Figure 2 displays the UV–vis absorption spectrum obtained at normal incidence for SR4, IP-S, and IP-Dip in the 190–1100 nm range. The other SR samples exhibited the same absorptive profile (Supporting Information, SI).

**Figure 3:**
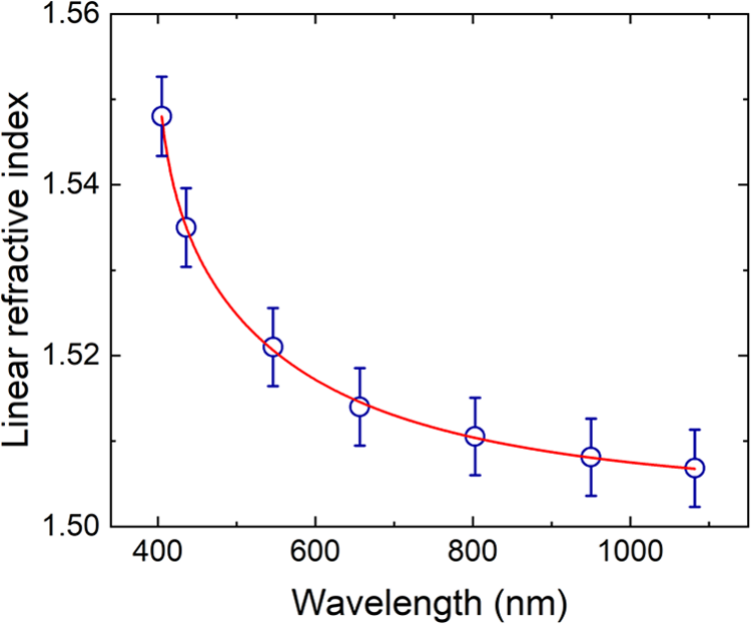
Linear refractive index as a function of the wavelength (open circles) for sample SR4. The red line corresponds to the Sellmeier fit.

We determined the linear refractive index of the samples measuring the critical angle in the visible range (400–700 nm) with an Abbe refractometer and the Fresnel reflection contribution in the transparent region (800–1100 nm) of the samples. The open circles in Figure 3 are the values of the linear refractive index as a function of the wavelength for SR4.

The results obtained for the other SR samples exhibited the same absorptive and refractive behavior (data not shown). The solid red line corresponds to the fit with the well-known Sellmeier equation with six parameters obtained with an iterative Levenberg–Marquardt algorithm
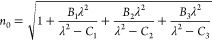
3where *n*_0_ is the linear refractive index, λ is the wavelength, and *B_n_* – *C_n_* (with *n* = 1, 2, 3) are the Sellmeier parameters. Fitting eq 3 yields parameters *B*_1_ = 6.0989 × 10^–1^, *C*_1_ = 2.095 × 10^–2^ μm^2^, *B*_2_ = 5.23 × 10^–3^, *C*_2_ = 1.4582 × 10^–1^ μm^2^, *B*_3_ = 6.424 × 10^–1^, and *C*_3_ = 1.73 × 10^–3^ μm^2^.

From the Sellmeier equation we can evaluate temporal and spatial dispersion effects that the polymers impose on a beam propagating through them. This is accounted for by the second-order dispersion, which relates the refractive index to the group velocity *v*_g_. In this case, the group velocity dispersion (GVD) or the group delay dispersion (GDD) per unit length is given by d(1/*v*_g_)d*ω* = d^2^(*n*_0_ω/*c*)/d*ω*^2^, where is *v*_g_ is the group velocity and ω is the frequency of the beam. In general, the GVD of these samples is relatively low. Table 2 shows the GVD at 780 nm for the polymers, silica, and SF5. We determined the GVD of IP-S and IP-Dip based on previously reported Sellmeier parameters.^37^

**Table 2:** Group Velocity Dispersion of All Polymer Samples, Silica, and SF5 Glass at 780 nm Determined by the Sellmeier Equations

sample label	GVD (fs^2^/mm)@780 nm
SR samples	77.8
IP-S^a^	65.6
IP-dip^a^	97.8
silica^b^	37.6
SF5^b^	131.5

a We calculated the GVD of IP-S and IP-Dip based on previously reported Sellmeier parameters.^37^

b The GVD for silica and SF5 are provided by the refractiveindex.info database of optical constants.

**Figure 4:**
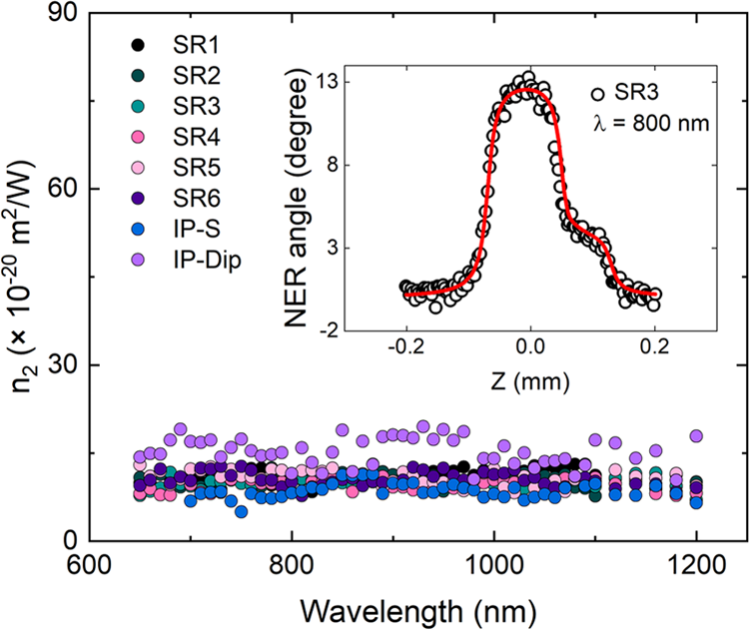
Nonlinear refractive spectra. Nonlinear refraction spectra of all samples determined by NER. Inset: NER curve for SR3 at 800 nm.

The nonlinear refractive dispersion of the samples was determined from the rotation angle using the NER technique. We used a tunable femtosecond pulse source in the 600–1200 nm spectral range. Because the *n*_2_ value depends on the initial polarization of the beam, we prepared elliptical polarization states to achieve the best signal-to-noise ratio of the NER angle. This was equivalent to setting the angle of the quarter-wave plate at 22–24°. The inset in Figure 4 displays a typical NER curve obtained for the polymer sample on top of silica (reference substrate), where the plateau and small shoulder correspond to the NER signal for polymer and silica, respectively. The theoretical fit (solid curve) of the experimental points is obtained using eq 1. The graph in Figure 4 shows the nonlinear refractive index as a function of wavelength for all samples.

The NER technique also enables the measurement of nonlinear absorption from the transmittance variation. The samples display variations at the noise level of the experiment, which sets an upper bound for the nonlinear absorption. In this case, we estimated the upper-bounded two-photon absorption coefficient β using eq 2.

The refractive and absorptive data allow us to understand the potential of these polymers for application as all-optical devices. To this end, we evaluated this potential using two quantifiers, *W* and FOM_2PA_, defined as^38,39^
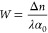
4and
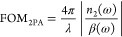
5where Δ*n* = *n*_2_*I* is the maximum refractive index change and all the other quantities were already defined in the text. Table 3 shows the average value of *n*_2_, β, *W*, and FOM_2PA_.

**Table 3:** Average Values of the Nonlinear Refractive Index *n*_2_, Upper-Bounded Two-Photon Absorption Coefficient β, and Figures of Merit *W* and FOM_2PA_ for All Polymers

sample label	*n*_2_ (×10^–20^) m^2^/W	β (×10^–14^) m/W	*W*	FOM_2PA_
SR1	11 ± 2	29 ± 6	120 ± 20	(2 ± 1)π
SR2	10 ± 2	13 ± 3	200 ± 40	(6 ± 3)π
SR3	10 ± 2	10 ± 2	140 ± 30	(6 ± 3)π
SR4	10 ± 2	12 ± 2	170 ± 30	(4 ± 2)π
SR5	11 ± 2	16 ± 3	110 ± 20	(3 ± 2)π
SR6	11 ± 2	27 ± 5	110 ± 20	(2 ± 1)π
IP-S	9 ± 2	21 ± 4	80 ± 20	(2 ± 1)π
IP-dip	16 ± 5	21 ± 4	50 ± 20	(4 ± 2)π

Due to the different methods of obtaining the parameters in Table 3, the averages have different meanings. The average *n*_2_ and *W* are mean scores of the spectral dependence based on the values of *n*_2_ and α_0_ as a function of wavelength. On the other hand, since β represents an upper bound on two-photon absorption, the average β is the mean score of a distribution of estimates at different wavelengths. Therefore, FOM_2PA_ values inherit the spectral dependence of *n*_2_, though not of β. The average FOM_2PA_ values are the mean over the spectrum, weighted by β estimates at each wavelength.

## 4 Discussion

The six SR samples in group I are acrylate-based photopolymerized resins. Acrylate-based compounds are extensively used in two-photon photopolymerization (TPP) experiments due to their optical quality^19^ and the possibility of easy functionalization with other materials.^40,41^ Moreover, their biocompatibility has been demonstrated with different organisms.^42,43^ Despite photoinitiators typically being regarded as toxic, we utilized Lucirin TPO-L in sufficiently small quantities to be nearly entirely consumed in the reaction.^43^ The use of a hybrid composition for samples SR1–6 is usually driven by the desirable mechanical properties conferred by each monomer. For example, the aromatic ring of the monomer SR 368 grants rigidity to the final compound, while the long chain of the monomers SR 499 and SR 399 grants flexibility and reduces the shrinkage intrinsic to the polymerization process.^44^ The molecular structure of each monomer can be seen in Figure SI2 (SI). While the study of mechanical properties is not the focus of our work, it is known that rigidity and flexibility are important properties for DLW applications ranging from biomimetics to photonic devices. It is a common practice to mix resins with different elastic moduli to tune the mechanical properties of the final structure.^45,46^ For example, in 3D biomimetics, using different polymers allows tuning of mechanical properties to replicate features of brain tissue and collagenous bone.^46^ In photonic devices like whispering gallery mode microresonators, the structural quality depends directly on the monomer ratio used, with the 70:30 (SR 368/SR 499) proportion shown to be optimal for these devices.^47^ Here, we observed that the different compositions do not seem to influence the optical properties that we have studied. All SR samples are transparent above 600 nm, while showing intense absorption around 400 nm, which is mainly associated with the photoinitiator.^48^ The samples present absorption spectra similar to the SR4 spectra in Figure 2(a). On the other hand, IP-S and IP-Dip Nanoscribe samples showed a more intense absorption region around 500 nm, which may also be related to the photoinitiators in their composition. These spectra are consistent with other previously reported investigations.^37^ It can also be seen that the IP-Dip sample exhibited non-negligible absorption up to around 900 nm. In contrast, the IP-S sample showed a small absorption band between 600 and 700 nm. Figure 3 shows the linear refractive dispersion spectrum for SR4 and the corresponding fit with the Sellmeier equation for *n*_0_. To avoid overestimation of *n*_0_ values due to scattering in the solid samples, we used the Abbe refractometry method for measuring *n*_0_ in the region most susceptible to scattering effects. In the infrared region, where scattering can be neglected, we used the Fresnel method with a UV–vis spectrometer. Within the precision of our measurements, we did not observe significant variations in the value of *n*_0_ of the SR samples, making the plot in Figure 3 representative of the SR samples. As observed in Figure 3, there is good agreement between the experimental data and the Sellmeier fit. In the higher transparency region, SR samples exhibited a linear refractive index of about 1.51. To our knowledge, this is the first time that a set of different proportions of the tri- and pentaacrylate SR monomers has its refractive properties characterized. All the polymers are appreciably similar, exhibiting comparable refractive indices and low GVD, which is closer to silica than to SF5 in Table 2. Furthermore, a comparison among the polymers shows that the GVD of the SR samples tends to fall between the IP-S and IP-Dip samples.

We obtained the *n*_2_ values by measuring the rotation angle in the NER experiment with the setup illustrated in Figure 1. The advantage of using this technique to extract *n*_2_ is 2-fold. Extracting nonlinearities via NER is more robust to certain experimental artifacts. Phase measurement is not prone to variations in transmittance induced by scattering, in contrast to techniques reliant on magnitude measurement alone. While NER retains most of the advantages of *Z*-scan, the introduction of a dual-phase lock-in amplifier into the setup depicted in Figure 1 provides the capability for accurate measurements of even small rotation angles. Furthermore, the limitation of NER in accessing only the *B* component of χ^(3)^ does not imply a loss of generality in our study. As previously reported, the ratio *B*/*A* depends on the physical origin of *n*_2_, and different techniques can access components *A* and *B* to discriminate this origin. In our study with ultrashort pulses and low repetition rates to characterize photopolymerized solids, orientational and thermal contributions to *n*_2_ can be neglected, and for a purely nonresonant electronic origin, *B*/*A* = 1. Therefore, the NER technique fully captures the nonlinear refractive behavior of the samples.

Figure 4 presents the results of the nonlinear refractive dispersion of the polymers. We used a 1 mm sample of fused silica for referenced acquisition in all cases. For this purpose, we considered 3 × 10^–20^ m^2^/W as the nonlinear refractive index of silica, which is consistent with the literature.^49−51^ As can be seen in Table 3, samples SR1–6 exhibited an average nonlinear refractive index of three to four times higher than fused silica, a widely used photonic material. These higher values are of even greater interest when compared to the constant behavior of the *n*_2_ dispersion and low GVD regardless of the monomer composition. This characteristic suggests excellent flexibility in applications with these organic materials, as refractive nonlinearities do not constrain the choice of desired mechanical properties for the polymers studied here. For IPS, Figure 4 also shows an approximately constant spectrum with average *n*_2_ equivalent to those of SR samples, within the experimental error. We measured the *n*_2_ of IP-S at wavelengths greater than 700 nm, far enough from the small band in the linear absorption spectrum shown in Figure 2(b) to avoid spurious effect on the nonlinear measurements. On the other hand, IP-Dip exhibited higher values, with an average *n*_2_ of more than five times the value for fused silica, as shown in Table 3. Although we can find a region with nonvanishing linear absorption in the spectral window of 600–1100 nm displayed in Figure 2(c), the *n*_2_ values were approximately wavelength-independent in that region, as show in Figure 4. Typically, absorption reduces the effective length of the material, contributing to an enhanced *n*_2_ value. This correction can be performed by determining the linear absorption coefficient and introducing *L*_eff_*=* (1 – e^–α*L*^)/α in eq 1. However, this correction had a negligible effect in our experiment, suggesting that absorption here does not play a significant role. Moreover, the reduced thickness of the IP-Dip sample compared to other sample does not introduce spurious variations in the *n*_2_ values obtained from eq 1. This general expression holds for materials whose thickness is much larger or smaller than the Rayleigh length of the incident Gaussian beam. Finally, the commercial formulation of IP-Dip includes various compounds, especially some photoinitiators, which may account for the higher *n*_2_.

The high nonlinearity and well-behaved dispersion of the samples point to the promising potential of organic photonic materials in shaping the next generation of devices. However, high *n*_2_ values alone do not guarantee the suitability of materials as nonlinear devices. Evaluating the prospect of different materials as photonic devices usually involves a comparative metric of the nonlinear response of interest against effects that introduce losses. For all-optical applications, the quantifier W in eq 4 compares the nonlinear refractive index against one-photon absorption-associated loss. We determined W for all samples from the *n*_2_ measurements and the linear absorption data. We observed a trend in Table 3 of SR1–6 samples achieving higher values, indicating greater robustness to one-photon absorption losses. Similarly, the quantifier FOM_2PA_ in eq 5 evaluates *n*_2_ against two-photon absorption-associated loss. Since the NER technique also allows for the determination of nonlinear absorption, we estimated the upper-bounded two-photon absorption coefficient for all samples in the 650–1200 nm window. The primary absorption of the samples is characterized by the nearly vertical absorption profile below 500 nm in Figure 2. However, Urbach states introduce an exponential absorption profile that decreases to around 600 nm. Such absorption allows two-photon-induced transitions with excitation up to about 1200 nm. Two-photon-induced transitions mediated by Urbach states are relatively unlikely and imply low values of β. Indeed, we did not observe nonlinear absorption in our experiment, suggesting that such absorption is below the detection sensitivity. Consequently, the noise level of the experiment provides an upper bound for nonlinear absorption in the samples, and the FOM_2PA_ values we determined represent a lower bound. In a scenario with lower noise and, if possible, higher sensitivity, the FOM_2PA_ values could be higher, indicating an even more suitable and interesting profile for nonlinear optical devices. All samples show comparable average FOM_2PA_ in Table 3, within the experimental error.

## 5 Conclusions

In summary, we have determined the linear and nonlinear absorption and refraction of eight different UV-photopolymerized resins commonly used in DLW. We obtained the refractive dispersion through a recently proposed NER method under femtosecond pulse excitation and also estimated the nonlinear absorption coefficient of all samples. Finally, we have discussed the suitability of the materials as photonic devices based on comparative metrics, such as GVD, W, and FOM_2PA_ of all samples.

We have shown that although different monomer proportions in the SR samples may offer mechanical advantages of interest for DLW, they do not affect the optical properties of the samples. The SR samples exhibit low GVD, with values falling between those of the IP-S and IP-Dip samples. On the other hand, all the samples are comparable regarding the average *n*_2_ values, within the experimental error. The polymers exhibit an average *n*_2_ three to four times higher than the *n*_2_ of silica, a widely used photonic material. However, IP-Dip shows a trend toward higher average values. The relatively high *n*_2_ of the polymers is particularly advantageous for nonlinear photonic devices, such as all-optical switches, where absorption-mediated losses can be a limitation. The average W values we found indicate greater robustness against one-photon absorption losses in the SR samples compared to the IP-S and IP-Dip samples. By contrast, the average lower-bounded FOM_2PA_ values indicate a trend of comparable robustness against two-photon absorption losses across all samples. Our results underscore the use of polymers in DLW, highlighting their optical versatility and corroborating the prospects of organic photonics.

## Supporting Information Available

Molecular structure of the SR monomers, and absorption spectra of the other SR samples (PDF)
