# Supplementary material for: Nonlinear Refraction and Absorption in Polymers Used for Femtosecond Direct Laser Writing
Source: ACS Omega. 2024 Dec 24;10(1):1440–7. doi: 10.1021/acsomega.4c09152 (PMC11739946; doi:10.1021/acsomega.4c09152)
Supplement: Supplementary file 1 — ao4c09152_si_001.pdf [file ao4c09152_si_001.pdf]

Nonlinear refraction and absorption in polymers used for femtosecond direct laser writing

Renan Cunha, João V. P. Valverde, Leonardo De Boni, Lino Misoguti, Cleber Renato Mendonça**

Instituto de Física de São Carlos, Universidade de São Paulo, São Carlos, SP, 13560-970, Brasil

Molecular structure of the three different acrylic resins used to prepare the SR samples

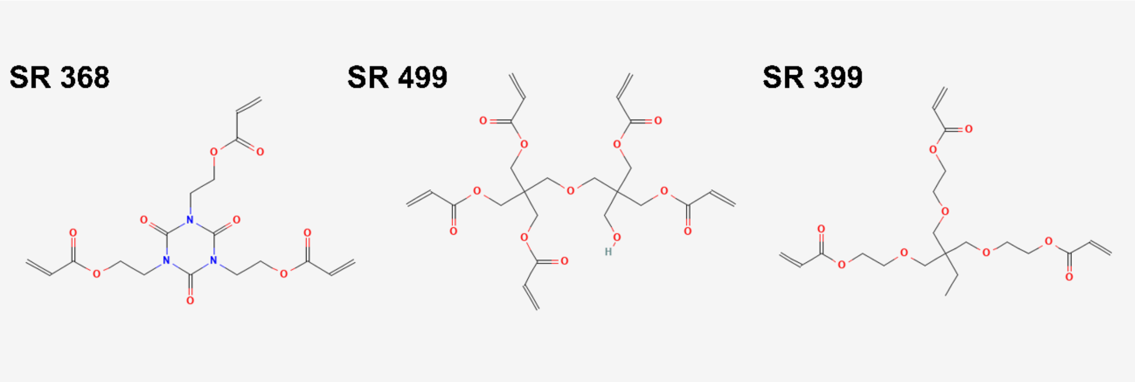

Figure SI1: Molecular structures of the Sartomer resins [1-3].

Absorption spectrum of the SR samples.

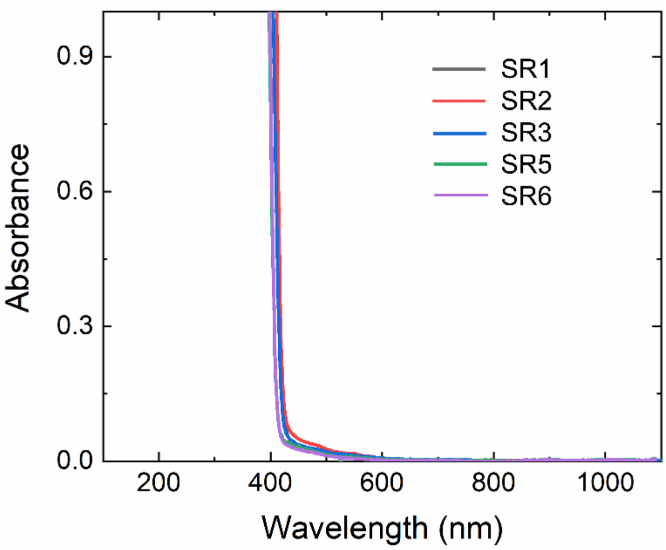

Figure SI2: Linear absorption spectrum of the SR samples. All samples exhibit a similar UV-Vis absorption profile.

REFERENCES

[1] National Center for Biotechnology Information (2024). PubChem Compound Summary for CID 170286, (2,4,6-Trioxo-1,3,5-triazinane-1,3,5-triyl)triethane-2,1-diyl trisprop-2-enoate. Retrieved November 20, 2024 from https://pubchem.ncbi.nlm.nih.gov/compound/170286.

[2] National Center for Biotechnology Information (2024). PubChem Compound Summary for CID 115157, (2-Ethyl-2-((2-((1-oxoallyl)oxy)ethoxy)methyl)-1,3-propanediyl)bis(oxy-2,1-ethanediyl) diacrylate. Retrieved November 20, 2024 from https://pubchem.ncbi.nlm.nih.gov/compound/115157.

[3] National Center for Biotechnology Information (2024). PubChem Compound Summary for CID 108956, Dipentaerythritol pentaacrylate. Retrieved November 20, 2024 from https://pubchem.ncbi.nlm.nih.gov/compound/Dipentaerythritol-pentaacrylate.
